# Metastatic Malignant Paraganglioma of Rare Sites Failed on Conventional Treatments Demonstrating Beneficial Response to ^177^ Lu-DOTATATE PRRT

**DOI:** 10.1055/s-0044-1791819

**Published:** 2024-10-25

**Authors:** Yeshwanth Edamadaka, Munita Bal, Swapnil Rane, Rahul V. Parghane, Sandip Basu

**Affiliations:** 1 Radiation Medicine Centre, Bhabha Atomic Research Centre, Tata Memorial Hospital Annexe, Parel, Mumbai, Maharashtra, India; 2 Radiation Medicine Centre, Homi Bhabha National Institute, Mumbai, Maharashtra, India; 3 Department of Pathology, Tata Memorial Hospital, Mumbai, Maharashtra, India

**Keywords:** paraganglioma, metastatic PGL, ^177^ Lu-DOTATATE peptide receptor radionuclide therapy, ^68^ Ga-DOTATATE PET-CT scan, sigmoid colon PGL, urinary bladder PGL

## Abstract

The incidence of paraganglioma (PGL) is rising due to better imaging modalities employed for evaluating incidentaloma and surveillance of the asymptomatic carriers. Benign and malignant PGLs often cannot be reliably diagnosed on histology alone, and the documentation of metastases is important in the diagnosis of malignancy. Advancement in genomics has improved our understanding of PGL. ^68^ Ga-DOTATATE positron emission tomography (PET)/computed tomography (CT) scan shows a significant superior detection rate compared with other conventional functional and anatomical imaging modalities, can detect rare sites of primary disease in PGL, and also aids in patient selection for peptide receptor radionuclide therapy (PRRT). PRRT is increasingly used in metastatic setting with good symptomatic and biochemical response and disease stabilization in metastatic PGL patients. We present a series of three patients with PGLs located in rare primary sites (sigmoid colon, urinary bladder, and carotid body space), which showed recurrence of disease on conventional treatments and developed metastatic disease in the lymph nodes, liver, skeleton, and lungs. PRRT with ^177^ Lu-DOTATATE achieved symptom control, favorable biochemical and imaging responses, and increased progression-free and overall survival rate in the described patients.

## Introduction

The increased incidence of paraganglioma (PGL) is probably a reflection of evolving indications for its evaluation, encompassing not only patients with signs and symptoms but also patients with incidentaloma and genetic causes or syndromes that require periodic surveillance. A significant advancement in genomics has improved our understanding of the hereditary foundation of PGL, further aiding in the development of individualized biochemical testing, imaging studies, therapeutic interventions, and follow-up. 1 We present a case series of three PGL located in rare primary sites along with metastases and their genetic association, pathological evaluation, biochemical analysis, and functional imaging, while also demonstrating the efficacy of peptide receptor radionuclide therapy (PRRT) in disease stabilization.

## Case 1

**Fig. 1:**
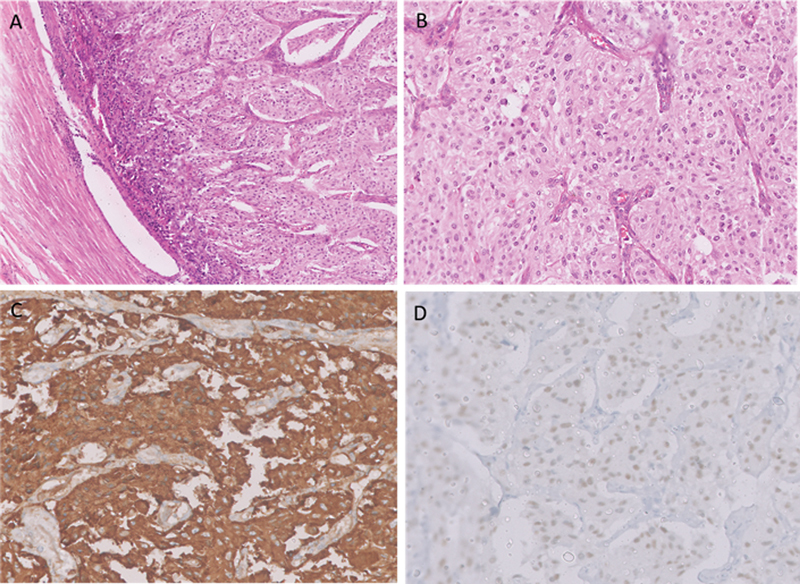
Histopathologic images of sigmoid colon paraganglioma (PGL). ( **A** ) Tumor with an organoid and nested architecture within the colonic muscularis propria. ( **B** ) The tumor is composed of uniform cells with salt and pepper chromatin and abundant eosinophilic cytoplasm. Immunohistochemistry images reveal ( **C** ) strong positivity for chromogranin and ( **D** ) diffuse nuclear GATA3 positivity, suggestive of sigmoid colon PGL.

**Fig. 2:**
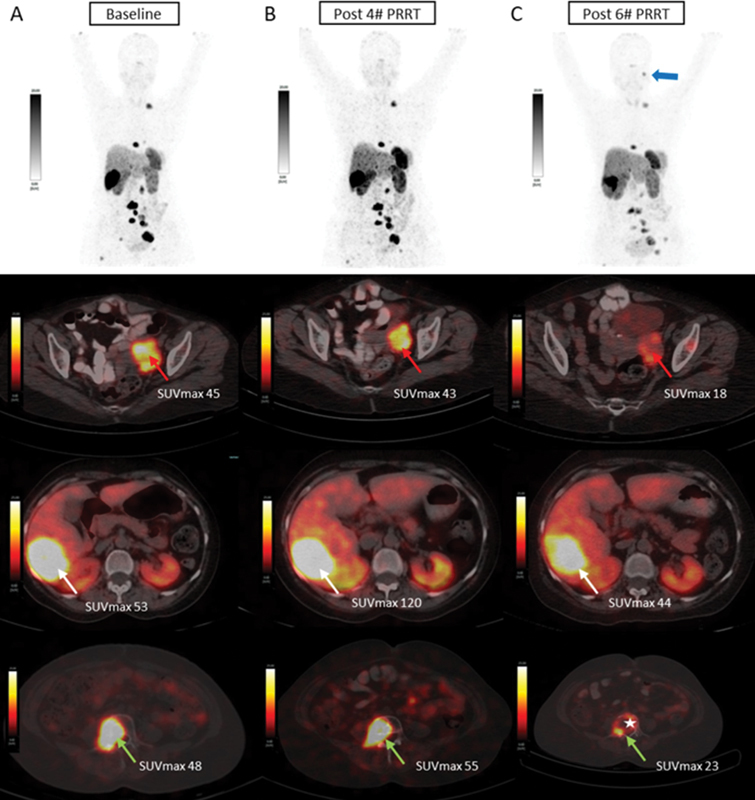
^68^ Ga-DOTATATE positron emission tomography (PET)/computed tomography (CT) scans at ( **A** ) baseline, ( **B** ) post four cycles of peptide receptor radionuclide therapy (PRRT), and ( **C** ) post six cycles of PRRT. Upper panel: maximum intensity projection (MIP) images; lower panel: fused axial images of PET/CT. Intense somatostatin receptor (SSTR) expression was noted in abdominopelvic nodes, dorsal and lumbar vertebral lesions, and a large segment VI hepatic lesion at baseline scan, post-four-cycle scan, and post-six-cycle scan. Stable disease was seen in ( **A** ) and ( **B** ). Post six cycles, heterogenous decreased SSTR expression was observed along with the appearance of new lesions in ( **B** ) and ( **C** ; *blue arrow* ). Axial fusion images (lower row) showed lumbar vertebral lesion (L4; *green arrow* ) at baseline ( **A** ), stable SSTR expression post four cycles ( **B** ), heterogenous decreased SSTR expression along with pathological compression fracture post six cycles ( *star* ; **C** ).

**Fig. 3:**
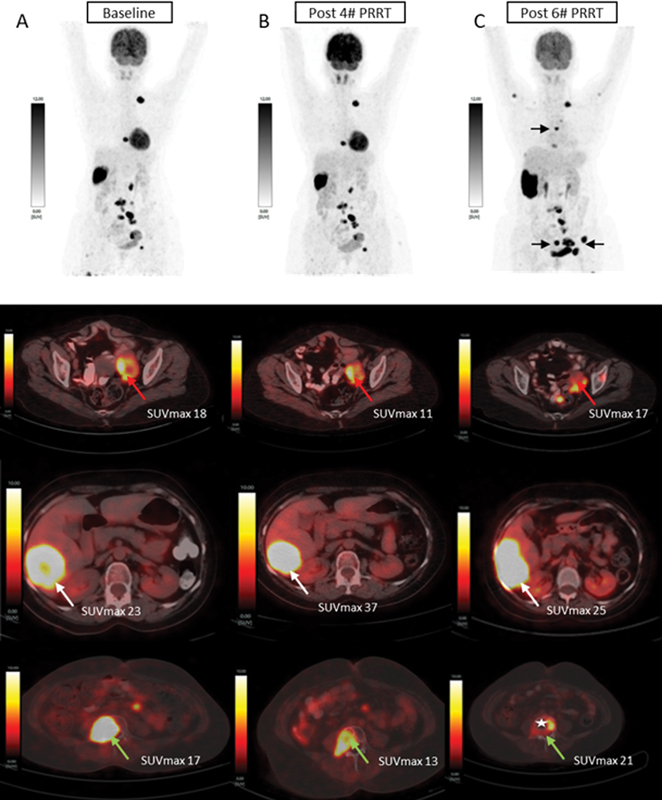
Fluorine-18 fluorodeoxyglucose ( ^18^ F-FDG) positron emission tomography (PET)/computed tomography (CT) scans at ( **A** ) baseline, ( **B** ) post four cycles of peptide receptor radionuclide therapy (PRRT), and ( **C** ) post six cycles of PRRT. Upper panel: maximum intensity projection (MIP) images. Lower panel: fused axial images of PET/CT. MIP images showed intense ^18^ F-FDG uptake in abdominopelvic nodes, dorsal and lumbar vertebral lesions, and a large segment VI hepatic lesion. PET/CT images ( **A, B** ) showed stable disease. Post six cycles, FDG-PET/CT scan ( **C** ) showed the appearance of new lesions ( *black arrow* ) suggestive of disease progression, and pathological compression fracture of the L4 vertebra was noted ( *star* ).

A 49-year-old female patient presented with abdominal pain and underwent computed tomography (CT), which showed a 4.4 × 3.4 cm enhancing polypoidal growth in the sigmoid colon with enlarged locoregional lymph nodes. She underwent sigmoid colon lesion and mesenteric lymph node resection. Histopathological analysis revealed monotonous tumor cells arranged in a zellballen pattern with hyperchromatic nuclei, evidence of necrosis, and mitotic figures along with metastatic nodal disease consistent with malignant PGL involving the sigmoid colon ( Fig. 1A, B ). After surgical resection, the patient was asymptomatic and developed new symptoms of backache 3 years later. A CT scan showed enlarged multiple preaortic and left iliac nodal masses suggestive of recurrence of PGL. Immunohistochemical analysis revealed the tumor cells were positive for chromogranin A and GATA3, but negative for cytokeratin (AE1/AE3). It also showed loss of succinate dehydrogenase B (SDH-B) expression ( Fig. 1C, D ). The patient underwent palliative chemotherapy (combination of vincristine, cyclophosphamide, and dacarbazine) and monthly depot octreotide long-acting repeatable injection. Follow-up CT scan showed bulky metastatic liver lesion measuring 8.7 × 5.0 × 7.2 cm in size, and the patient complained of increasing back pain. She underwent magnetic resonance imaging (MRI), which revealed an enlarged retroperitoneal lymph node and metastatic lumbar vertebral lesions. For these bony lesions, she was treated with palliative radiotherapy to the spine, which resulted in modest improvement in symptom. The patient was worked up for PRRT; at this time, she complained of palpitations and was on antihypertension medications with elevated plasma-free normetanephrine (PFNM) of 8,585 pg/mL at baseline; her ^68^ Ga-DOTATATE PET/CT scan showed intense somatostatin receptor (SSTR) uptake in metastatic enlarged abdominal and pelvic node lesions, multiple skeletal lesions, and a bulky hepatic lesion (segment VI) as shown in Fig. 2A . For further characterization of these lesions, the patient underwent fluorine-18 fluorodeoxyglucose ( ^18^ F-FDG) PET/CT scan, which showed intense ^18^ F-FDG uptake in concordant lesions as shown in Fig. 3A , which was expected **as she showed loss of SDH-B expression on immunohistochemistry (IHC)** . The patient was planned for combined therapy of ^177^ Lu-DOTATATE PRRT and chemotherapy of capecitabine and temozolomide (CapTem). After two cycles of PRRT and CapTem, the patient showed improvement in her baseline symptoms with improved performance status and her PFNM showed a slight reduction to 7,200 pg/mL. She received two more cycles of the same combined therapy. After four cycles, she became symptom free and her PFNM value was 36.59 pg/mL, suggestive of a favorable response to combined therapy. Dual tracer PET/CT evaluation showed stable disease on comparison as shown in Figs. 2A , B and 3A, B . She further received two more cycles. After six cycles of PRRT, she complained of low back pain and her PFNM elevated to 444 pg/mL. Dual tracer PET/CT evaluation showed new peritoneal, skeletal lesions (D5 vertebra, left acetabulum) and increase in the size of the hepatic lesion, suggestive of disease progression as shown Figs. 2B, C and 3B, C . She underwent external beam radiotherapy (EBRT) to the lumbar vertebra for symptomatic control. Later on, she developed hemiparesis due to compression fracture of the L4 vertebra.

## Case 2

**Fig. 4:**
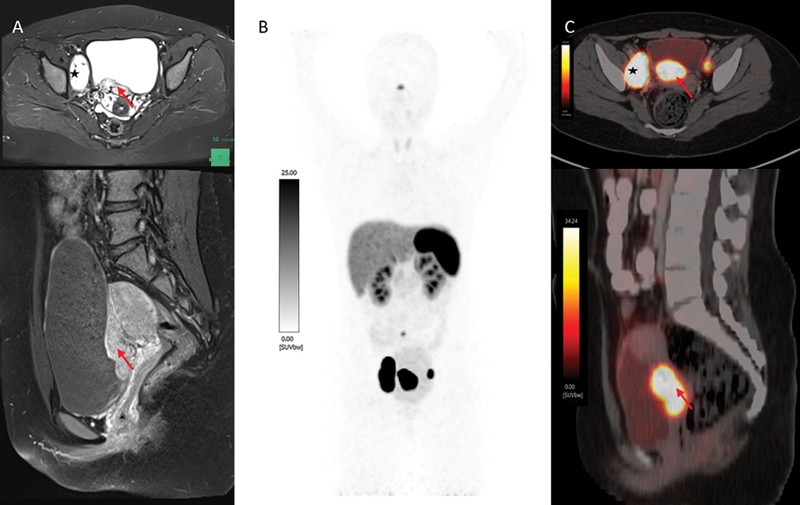
( **A** ) Magnetic resonance imaging (MRI) and ^68^ Ga-DOTATATE positron emission tomography (PET)/computed tomography (CT) scan at baseline. Axial MRI (upper) and sagittal (lower) images showed ill-defined short tau inversion recovery (STIR) hyperintense lesion with postcontrast enhancement ( *red arrow* ), focal infiltration of the bladder wall, and enlarged pelvic nodal masses ( *star* ) showing similar characteristics to the primary lesion. ( **B** ) Maximum intensity projection (MIP) image and ( **C** ) axial and sagittal images showed increased somatostatin receptor (SSTR) expression in hyperenhancing lesion in the posterior and posterolateral walls of the urinary bladder ( *red arrow* ) and metastatic iliac nodes ( *star* ; right > left).

**Fig. 5:**
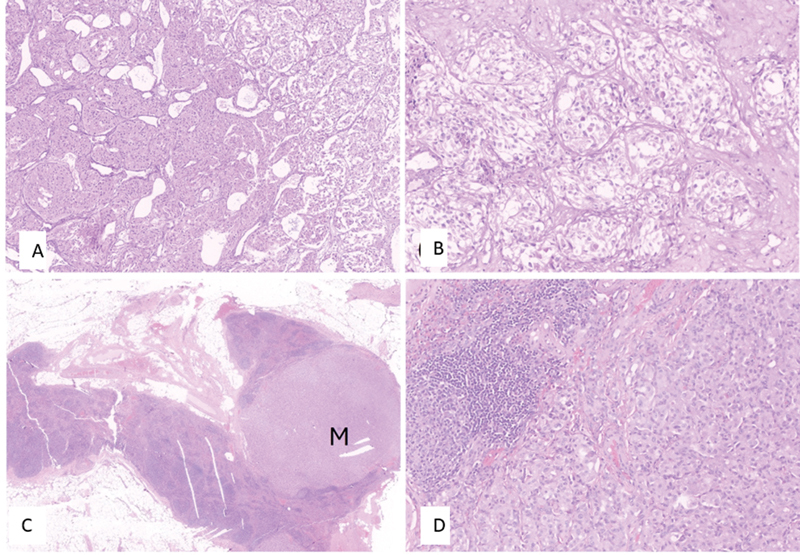
Histological images of a urinary bladder paraganglioma (PGL). ( **A** ) Images showed the nesting and zellballen architecture of the tumor in 100x optical magnification, hematoxylin and eosin (H&E) stain. ( **B** ) The 200x optical magnification of H&E-stained slide image showed the cellular details with eosinophilic to vacuolated cytoplasm of the tumor cells with tumor cells showing conspicuous nucleoli. ( **C** ) The lymph node lesion images showed metastases (M) of the same tumor in H&E stain, 20x optical magnification. ( **D** ) High magnification (200x) of the same nodal metastases with similar tumor morphology as seen in the urinary bladder tumor, suggestive of urinary bladder PGL with lymph nodal metastases.

**Fig. 6:**
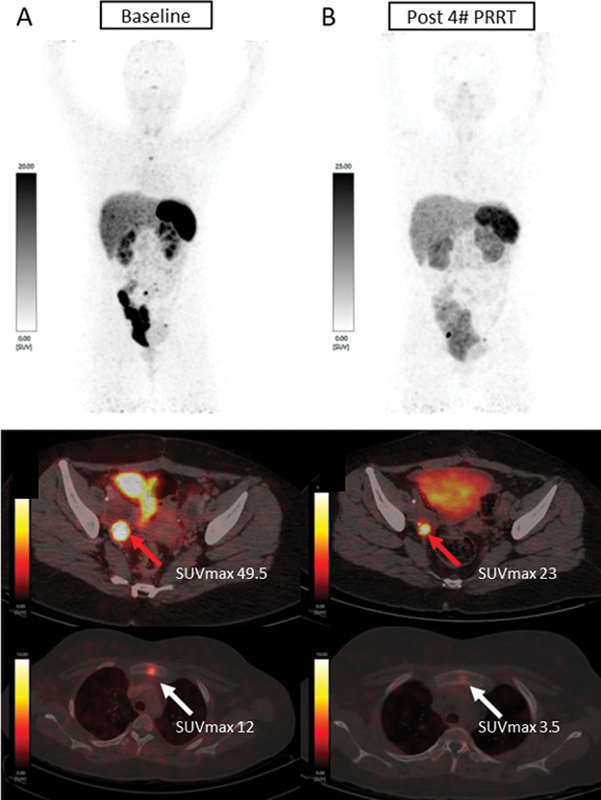
^68^ Ga-DOTATATE positron emission tomography (PET)/computed tomography (CT) scans at ( **A** ) baseline and ( **B** ) post four cycles of peptide receptor radionuclide therapy (PRRT). Upper panel: maximum intensity projection (MIP) images; lower panel: fused axial images of PET/CT. Increased somatostatin receptor (SSTR) expression was seen in enlarged pelvic nodes and sclerotic sternal lesion at baseline ( **A** ). Post four cycles of PRRT, PET/CT images showed decreased SSTR expression and size of pelvic lymph nodes and sternal lesion. SUVmax, maximum standardized uptake value.

A 15-year-old adolescent girl presented with complaint of painless hematuria for 1 month. Cystoscopy showed a solid highly vascular mass in the posterior wall of the urinary bladder; her MRI revealed an intramural T2 hyperintense lesion in the right half of the bladder base measuring 2 × 2 ×4.6 cm and T2 hyperintense lesion in the bilateral pelvic nodes measuring 4.5 × 2.5 cm suggestive of metastatic PGL as shown in Fig. 4A , and her urinary catecholamine level was elevated (300 pg/mL). The patient underwent ^68^ Ga-DOTATATE PET/CT scan, which showed intense SSTR expression in the urinary bladder lesion and the pelvic lymph nodes as shown in Fig. 4B, C . She underwent uterus-, ovary-, and vagina-sparing radical cystectomy with formation of Hautmann's neobladder. Histology revealed a PGL infiltrating the lamina propria and detrusor muscle with overlying urothelium free of tumor and metastatic PGL in locoregional nodes shown in Fig. 5 . Postsurgery, her PFNM was 5.84 pg/mL. On follow-up, her PFMN levels (165 pg/mL) and urinary vanillylmandelic acid (VMA) (136.6 mg/g) was elevated. A CT scan showed a 2.1 × 1.0 cm intensely enhancing right internal iliac node and right common iliac node. Her ^68^ Ga-DOTATATE scan showed new SSTR expressing sclerotic sternal and calvarial lesions as shown in Fig. 6A . She underwent two cycles of ^177^ Lu-DOTATATE PRRT and on response evaluation showed reduced biochemical markers with PFMN (46.74 pg/mL) and urinary VMA (4.01 mg/g) and stable disease on imaging. She received two more cycles of PRRT. After four cycles, her PFMN was raised to 62.9 pg/mL, but imaging evaluation showed no new lesions, suggestive of stable disease as shown Fig. 6B . During her routine annual follow-up, the imaging evaluation showed no new lesions, suggestive of disease stabilization. The patient is alive and doing well on regular follow-up at 36 months after her first PRRT.

## Case 3

**Fig. 7:**
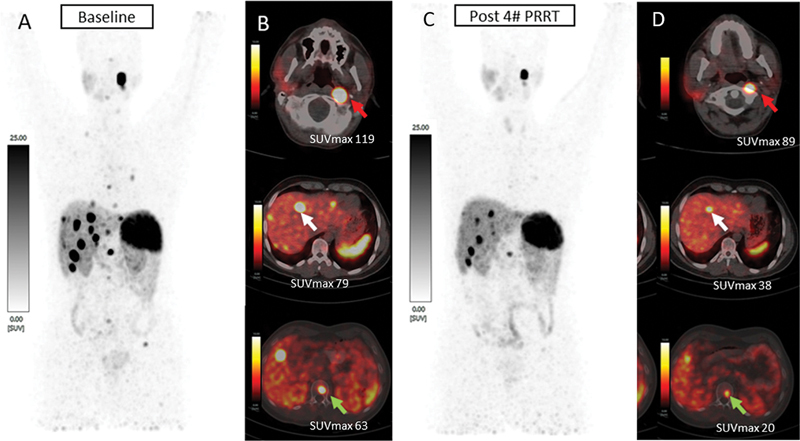
^68^ Ga-DOTATATE positron emission tomography (PET)/computed tomography (CT) scans at ( **A, B** ) baseline and ( **C, D** ) post four cycles of peptide receptor radionuclide therapy (PRRT). Maximum intensity projection (MIP) image and fused axial images of PET/CT scan showed intense somatostatin receptor (SSTR) expression in residual carotid body bladder paraganglioma, intense SSTR expression in multiple hepatic lesions, and skeletal lesions at baseline. Post four cycles of PRRT, the MIP and fused axial images of PET/CT scan showed a reduction in SSTR uptake, size and number of carotid body lesion, and hepatic and skeletal lesions suggestive of a favorable response to PRRT. SUVmax, maximum standardized uptake value.

A 24-year-old male patient presented with increasing left-sided neck swelling and was evaluated with contrast-enhanced MRI, which showed a carotid body lesion. He underwent incomplete resection of the carotid body lesion with carotid diversion endarterectomy-anastomosis of the internal carotid artery. Histology revealed a carotid body tumor and he was treated with adjuvant radiotherapy to the neck. His biochemical markers were normal, suggesting a nonsecretory PGL. He had only residual tumor on follow-up evaluation. Two years after surgery, he had recurrence in the left cervical region along with multiple contrast-enhancing lesions in the liver and bilateral lung nodules. ^68^ Ga-DOTATATE PET/CT scan showed intense SSTR uptake in the residual carotid body lesion, multiple liver lesions, and sclerotic skeletal lesion with lung nodules as shown in Fig. 7A, B . Subsequently, the patient received four cycles of ^177^ Lu-DOTATATE PRRT. After four cycles of PRRT, ^68^ Ga-DOTATATE PET/CT scan showed decreased SSTR expression in the residual carotid and liver lesions, and decreased in number and size of liver lesions, suggestive of a favorable response to PRRT as shown in Fig. 7C, D . On last follow-up, the patient is alive and symptom free, with no new lesion appearance on ^68^ Ga-DOTATATEPET/CT scan, suggestive of partial response with progressive-free survival (PFS) of 36 months after the first PRRT cycle.

## Discussion

PGL is a rare nonepithelial neuroendocrine tumor (NET), which tend to occur along the sympathetic and parasympathetic ganglia, sometimes from neuronal innervations. PGLs have the highest known hereditable risk among cancers; hence, genetic testing is critical not only for the patient but also for relatives who may harbor the same germline mutation. Late age of onset or lack of family history of PGL does not exclude a germline mutation, which could be attributable to low penetrance of some gene mutations. The most prevalent PGL driver mutations include SDH-B, SDH-D, VHL, RET, and neurofibromatosis type 1 (NF1). 2 The diagnosis of functioning PGL using plasma-free metanephrines offers a high sensitivity of 99% and an intermediate specificity of 89% with the highest specificity offered by urinary vanillylmandelic acid. 3 PGLs have a varied risk of malignancy; approximately 15 to 20% of these tumors are metastatic. Because no histological, genetic, or molecular markers can reliably distinguish between benign and malignant diseases, the occurrence of metastases is used to diagnose malignancy. Metastatic PGLs are defined by the presence of metastatic lesions in unanticipated neural tissue locations such as the lymph nodes, bones, the lung, or the liver. 4

PGLs with SDH enzyme complex II mutations comprise of more than 40% of the total metastatic PGL and 10% of the total mutation burden of PGL. The SDH-B gene mutation was highly correlated with extra-adrenal manifestations and early onset of PGL, as well as with recurrence and malignancy. 5 SDH enzyme is comprised of four subunits A, B, C, or D; any alteration in any of the SDH genes lead to loss of SDH enzymatic function and absence of SDH-B expression. SDH-B immunohistochemistry (IHC) can detect the majority of SDH subunit mutations, and is a useful tool to detect SDH mutation tumors. 6 SDHB/SDHA IHC with negative or weak diffuse staining is a reliable tool to identify patients with SDHx mutations. 7 The specificity of SDH-B staining is low in carotid PGL; nevertheless, its high sensitivity could be used for identification of patients carrying SDHx variants. 8 Succinate accumulation in SDH-deficient PGL acts as a competitive inhibitor of 1-oxoglutarate-dependent hypoxia-inducible factor (HIF) prolyl-hydroxylases stabilizing HIF-1α, facilitating angiogenesis and anaerobic metabolism. 9 The hypermethylator phenotype found in SDH mutations explains their tumor-suppressive role; risk of malignancy may be due to a severe epigenetic silencing of genes involved in cell differentiation. 10 The mean age at diagnosis was 29 years with prevalence of mutation carriers in population-based registries between 4 and 6%. Head and neck PGLs were statistically seen more in SDH-D carriers compared with SDH-B mutations, which are prevalent in intra-abdominal extra-adrenal tumors, although malignant tumors are more frequent in SDH-B mutations. 11 ^68^ Ga-DOTATATE showed a significantly superior detection rate compared with other functional and anatomical imaging modalities in the evaluation of SDH-B-related metastatic PGLs. 12

Metastatic sigmoid PGLs have not been reported in the literature to our knowledge. Tampakis et al 13 incidentally found a small-sized asymptomatic PGL in the diverticulum of the sigmoid colon measuring 0.6 mm, which was SDH deficient. Kimura et al 14 found a submucosal tumor in the sigmoid colon measuring 2.5 cm while evaluating for lower abdominal pain along with gastrointestinal stromal tumor as a part of the Carney–Stratakis dyad. A proposed The Grading of Adrenal Pheochromocytoma and Paraganglioma (GAPP) histological grading, which in these patients had well-differentiated histology and demonstrated no metastasis or disease recurrence on long-term follow-up, could be a prognostic factor. Hamidi et al 15 found that larger primary tumors were related with rapid disease progression and a greater mortality risk in 272 patients studied for over 55 years. To our knowledge, no metastatic sigmoid PGL with SDH-B deficiency has been reported, and we demonstrated the clinical benefits of PRRT with disease stabilization on clinical and biochemical evaluation with stable disease on imaging. However, eventually the patient experienced disease progression and died. Patients with SDH-deficient PGLs have a variable course ranging from indolent to highly malignant, implying a comprehensive approach to patient prognosis that includes tumor size, pathological grading, and TNM staging.

Urinary bladder PGL originates from the sympathetic nervous system embedded in the muscle layer of the bladder wall, accounting for 0.06% of all bladder tumors and 6% of extra-adrenal PGL. 16 Urinary bladder PGL presents with various clinical symptoms ranging from paroxysmal hypertension, headaches, and palpitations triggered by micturition leading to catecholamine excess to painless hematuria with or without dizziness. 16 Imaging findings include hypoechoic lesions with significant vascularity on sonography, arterial intramural hyperenhancing lesions on CT scan, and T2 hyperintense lesions on MRI. On cystoscopy, it appears as a yellow, submucosal tumor with no mucosal involvement unlike transitional urothelial carcinoma raising a suspicion. 17 Surgery is the standard of care including transurethral resection of bladder tumor, partial cystectomy, and radical cystectomy. 18 Synchronous metastasis is associated with increased disease-specific mortality and metachronous metastasis was associated with younger age, and developed at a median of 4 years after initial diagnosis. SDH-B association was observed in half of the tested patients in a cohort of bladder PGLs. 19 Ko et al 20 reported a synchronous metastatic bladder PGL in a 59-year-old patient with intense SSTR expression in bladder mass, local pelvic nodes, multiple bone and lung metastases who received PRRT postsurgery as a systemic treatment.

Head and neck PGLs arise from the parasympathetic nervous system. These are usually nonfunctional and rarely malignant. Carotid PGLs are the most common among them, and they usually present as a slow-growing mass in the anterior neck region. Malignant carotid PGLs are associated with poor prognosis with a 11.8% survival chance at 5 years. 21 Investigations to confirm the diagnosis include duplex ultrasound, a very sensitive and specific modality, CT angiography to determine its relationship to bony landmarks, MRI and MR angiography to establish possible vascular involvement, and arteriography as a gold standard. 22 The Shamblin classification system based on the tumor size and carotid artery involvement is mainly used to assess and establish risk of cranial nerve injury and intraoperative blood loss. 23 ^68^ Ga-DOTATATE-PET/CT identified more lesions than ^18^ F-FDOPA PET/CT and ^18^ F-FDG PET/CT in the localization of head and neck PGLs with potential implications for PRRT treatment. 24

The chemotherapy protocol in metastatic PGLs is based on the Averbuch protocol with cyclophosphamide, vincristine, and dacarbazine (CVD), but it fails to produce remission and is associated with significant marrow toxicity. 25 A retrospective series reported similar safety profile and efficacy using temozolomide in SDH-B mutations. 26 PRRT was evaluated in a mixed cohort of 15 PGL patients with unresectable and metastatic cases, with a median of three cycles administered. PRRT demonstrated a significant response with median PFS was not attained in head and neck PGL patients over a median follow-up of 27 months. The requirement of anti-hypertensives decreased in six of nine patients and along with decrease in PFNM levels. Response evaluation on the Response Evaluation Criteria in Solid Tumours 1.1 (RECIST 1.1) showed objective response rate in 80% of patients. 27 An interim analysis of a phase II study showed a longer PFS of 24.6 months in sporadic PGLs compared with 13.2 months in SDHx mutations, overall demonstrating high efficacy and safety profile for PRRT. 28

Similarly, another retrospective study showed a favorable response to ^177^ Lu-DOTATATE PRRT with minimal low-grade and easily manageable side effects in PGL patients with disease control rate of 67%, estimated PFS rate of 63% (95% confidence interval [CI]: 30–96%) and overall survival rate of 65% (95% CI: 32–97%) at 40 months after first cycle of PRRT. 29

To summarize, case 1 was an advanced metastatic sigmoid colon PGL. After surgical resection of the primary lesion, the patient developed recurrence and underwent six cycles of PRRT and CapTem (the combined chemo-PRRT approach) as described in gastroenteropancreatic (GEP) NETs. 30 The presently described case was first among PGLs where a combined therapy approach was used with initially good symptomatic control correlating with biochemical response. Subsequent follow-up showed progressive disease and resulted in a pathological fracture to the L4 vertebra causing hemiparesis, which was managed conservatively. She survived for 40 months after the start of the PRRT. Case 2 was an advanced metastatic urinary bladder PGL. After surgical resection of the primary lesion, the patient presented with evidence of biochemical and imaging recurrence, and she underwent four cycles of PRRT alone. She showed partial response on ^68^ Ga-DOTATATE-PET/CT scan and is alive with regular follow-ups. Case 3 was an advanced metastatic carotid body PGL. The patient initially had an incomplete resection and received local radiotherapy, but developed metastatic disease 2 years later. The patient underwent four cycles of PRRT alone, showing partial response of the liver metastases on imaging. Currently, the patient is alive and on regular follow-up at our institute.

## Conclusion

PGLs are detected at unusual locations, including the sigmoid colon, urinary bladder, and carotid body space. These rare site PGLs may exhibit metastatic disease, which may be challenging to manage due to the high rate of disease recurrence on conventional therapy. The described malignant metastatic PGL cases demonstrated high SSTR expression. Therefore, ^68^ Ga-DOTATATE-PET/CT imaging can be utilized in the assessment of these patients, which helps in detecting metastatic diseases in the lymph nodes, liver, skeletal, and lung regions, and also serves as potential application of theranostics. PRRT is an effective treatment for GEP NET and may be considered a useful therapeutic option for metastatic PGL patients. PRRT may result in a biochemical response, partial response/disease stabilization on imaging response, improved symptoms, and a prolonged PFS and overall survival in metastatic malignant PGLs located in rare primary sites.
